# Supplementary material for: Somatic CpG hypermutation is associated with mismatch repair deficiency in cancer
Source: Mol Syst Biol. 2024 Jul 18;20(9):2. doi: 10.1038/s44320-024-00054-5 (PMC11369196; doi:10.1038/s44320-024-00054-5)
Supplement: Supplementary file 9 — Expanded View Figures [file 44320_2024_54_MOESM9_ESM.pdf]

# Expanded View Figures

**Figure EV1. P-MACD CpG mutation load estimates in HM tumours and MMR gene mutations in CpG hypermutated tumours.**

(**A**) %nCg (with C > T) mutation load (min. estimate) derived for 1938 hypermutant (HM)-tumours using the P-MACD pipeline and stratified by tumours with somatic CpG hypermutator status (HM;CpGlo, $n = 76$ and HM;CpGhi, $n = 1860$). See Methods for more details about the nCg model. (**B**) nCg vs rCg (r = A or G) min. mutation load estimates from P-MACD in CpG hypermutated tumours. See Methods for more details about the nCg and rCg model. (**C**) The distribution of mutation consequences for deleterious genomic alterations observed in core mismatch repair genes within CpG hypermutated samples. (**D**) Theoretical proportion of non-synonymous mutations (vertical axis) for each dinucleotide context (horizontal axis) based on codon usage for each amino acid, showing no bias towards non-synonymous mutation of codons in C>TpG context (orange colour). For boxplots, the black central band represents the median. The lower and upper hinges represent the first and third quartiles, respectively. The whiskers represent the 1.5× interquartile range.

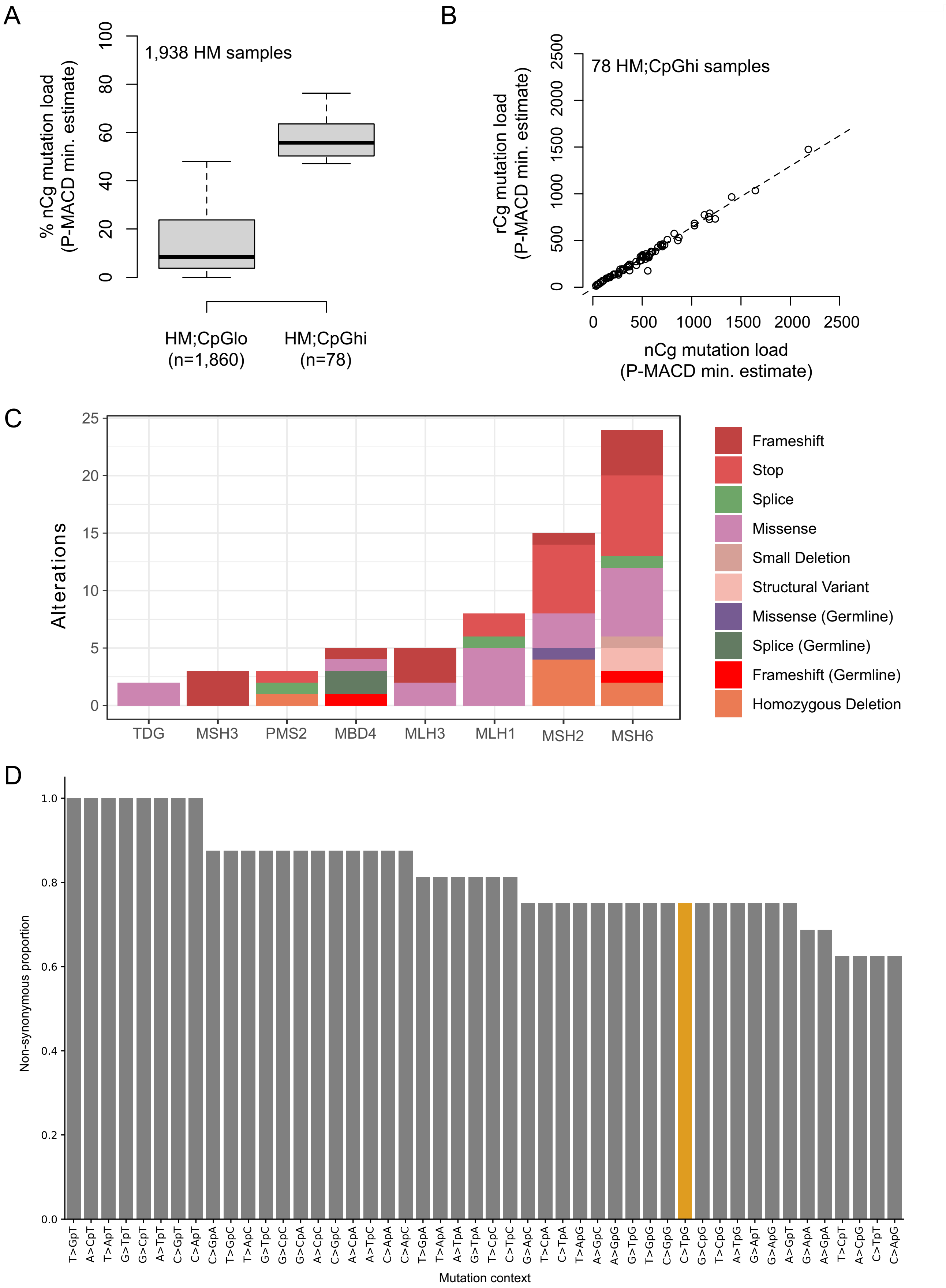

A
1,938 HM samples
% nCg mutation load
(P-MACD min. estimate)
0
20
40
60
80
100
HM;CpGlo
(n=1,860)
HM;CpGhi
(n=78)
B
78 HM;CpGhi samples
rCg mutation load
(P-MACD min. estimate)
0
500
1000
1500
2000
2500
nCg mutation load
(P-MACD min. estimate)
C
Alterations
0
5
10
15
20
25
TDG
MSH3
PMS2
MBD4
MLH3
MLH1
MSH2
MSH6
Frameshift
Stop
Splice
Missense
Small Deletion
Structural Variant
Missense (Germline)
Splice (Germline)
Frameshift (Germline)
Homozygous Deletion
D
Non-synonymous proportion
0.0
0.2
0.4
0.6
0.8
1.0
T>GpT A>CpT T>ApT G>TpT G>CpT A>TpT C>GpT C>ApT C>GpA T>GpC T>ApC G>TpC G>CpC G>CpA A>CpC C>GpC A>CpA A>TpC C>ApA C>ApC T>GpA T>ApA A>TpA G>TpA T>CpC C>TpC G>ApC T>CpA C>TpA T>ApG A>GpC A>GpG G>TpG T>GpG C>GpG C>TpG G>CpG T>CpG A>TpG G>ApT G>ApG A>GpT G>ApA A>GpA T>CpT A>CpG C>TpT C>ApG
Mutation context

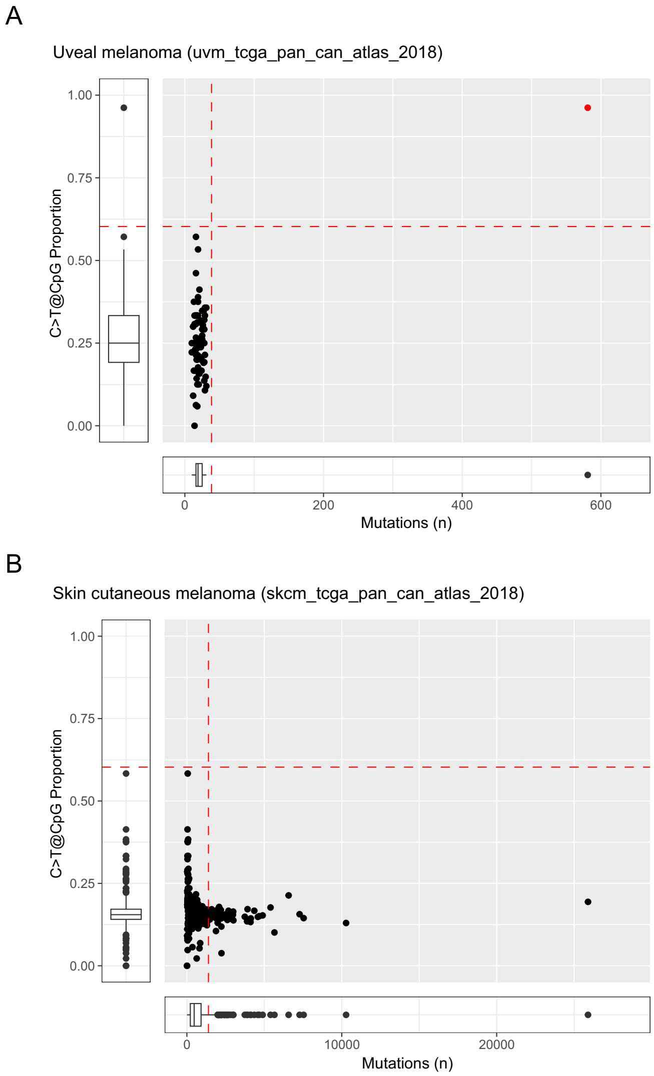


**Figure EV2. Outlier analysis of skin and uveal melanoma.**

Outlier analysis was performed on the 2018 TCGA (**A**) skin cutaneous melanoma ($n = 406$) and (**B**) uveal melanoma ($n = 80$) cohorts by applying Tukey's fence to identify high mutation load tumours and the cohort-wide cutoff for C > T@CpG proportion (see Fig. 1). Tumours exceeding both thresholds were considered CpG-Hypermutators (red dots). For boxplots, the black central band represents the median. The lower and upper hinges represent the first and third quartiles, respectively. The whiskers represent the 1.5× interquartile range.

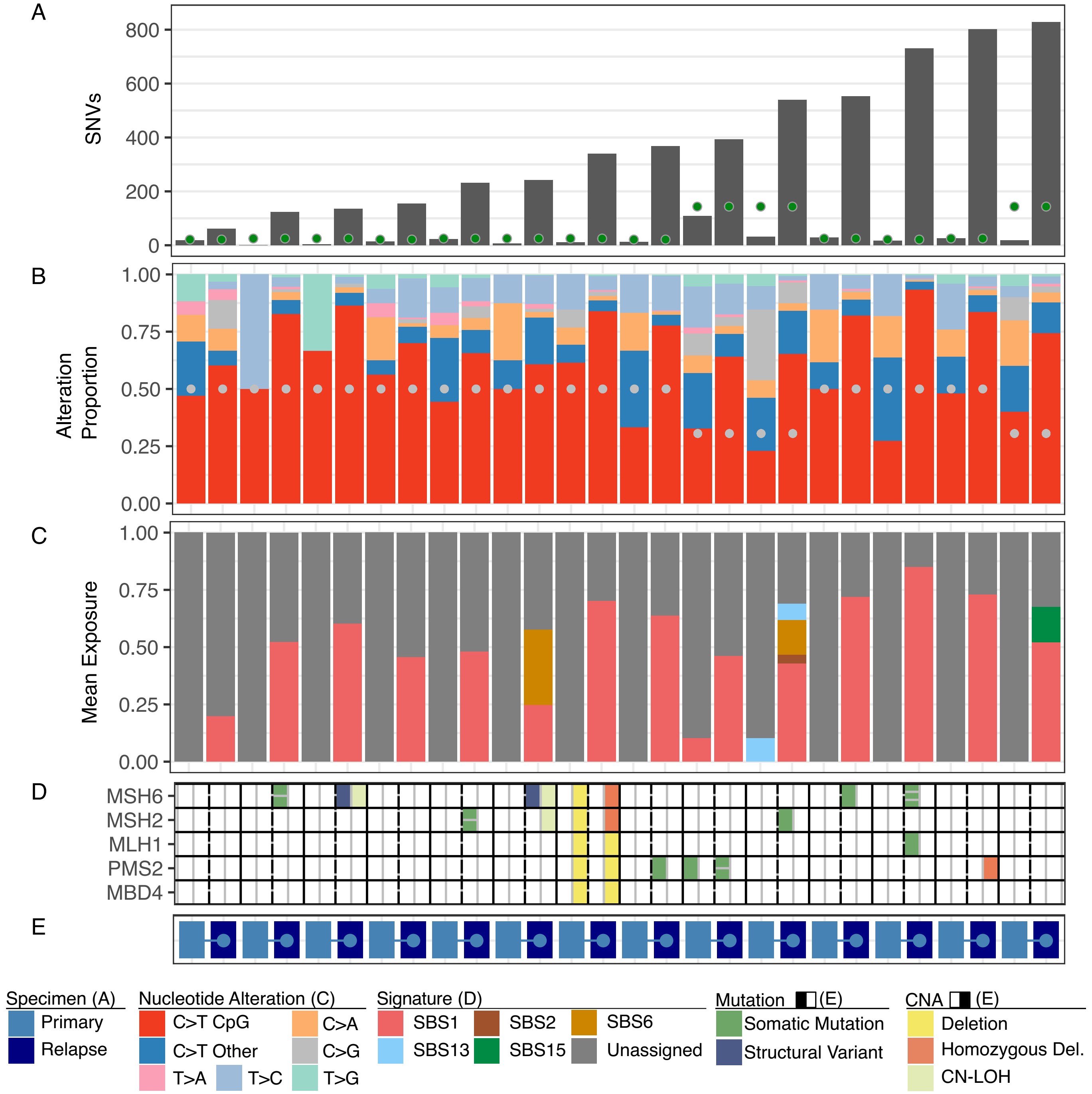


**Figure EV3. Relapse-specific somatic CpG hypermutation in paediatric ALL.**

Outlier analysis identified somatic CpG hypermutation in 14 paediatric acute lymphoblastic leukaemia. (**A**) The total number of somatic mutations found in each specimen. The 75th percentile for paediatric ALL in the cohort is marked by green dots. (**B**) The relative proportion of each of the six transition and transversion events, the proportion of C > T mutations were further divided into those occurring in a CpG context and in other contexts. Grey dots mark the 75th percentile for the proportion of mutations in a CpG context for paediatric ALL in the cohort. (**C**) Mean signature exposure was determined using the sigfit algorithm and COSMIC v3 mutational signatures. Signature assignment was not possible in many primary specimens due to low mutation load. (**D**) Genetic lesions (left column) and copy number alterations (right column) in members of the MMR pathway. (**E**) Mutation data were available from samples taken at the initial presentation (light blue), and after relapse (dark blue) Samples from a single patient are indicated by a linking line.

 © The Author(s)

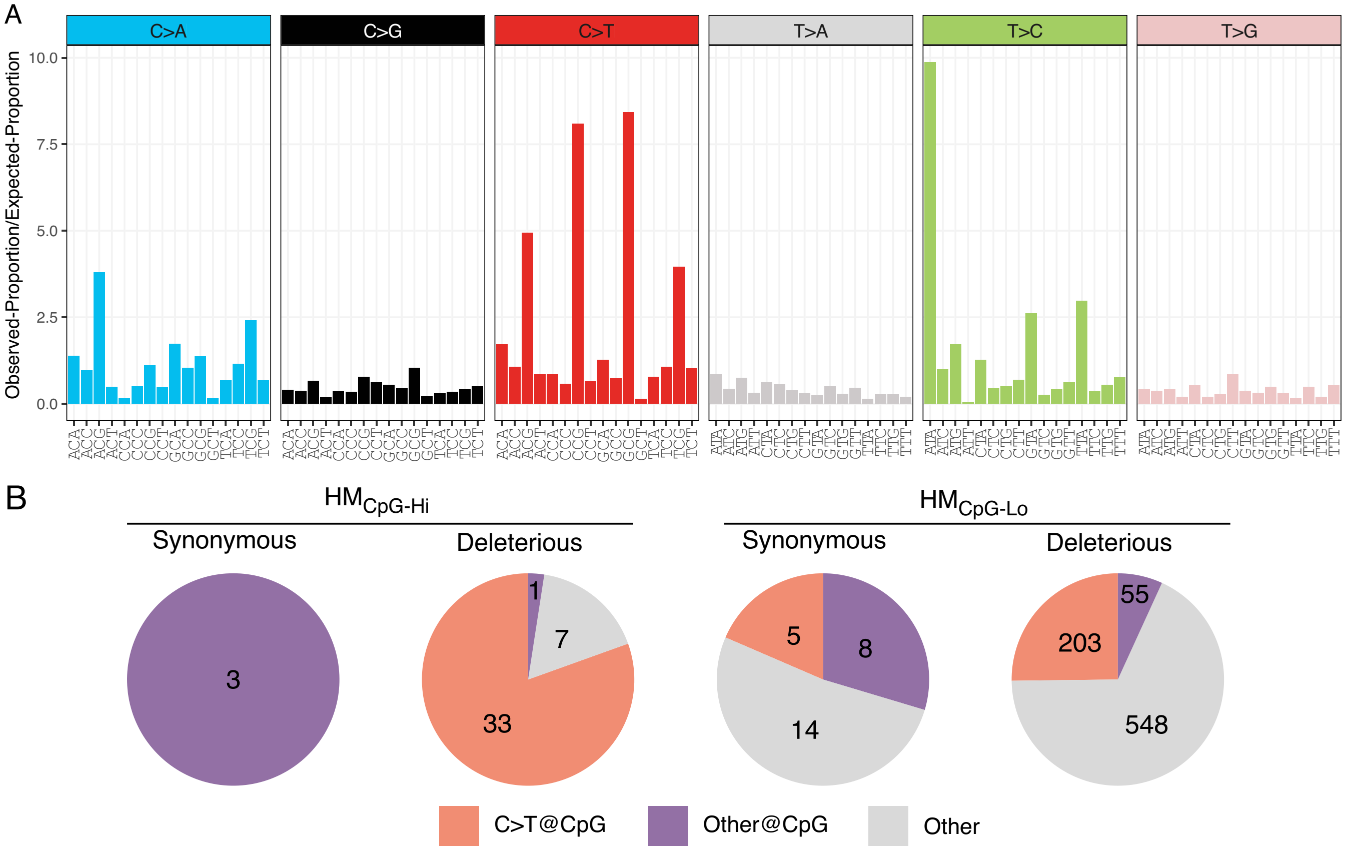


**Figure EV4. Somatic CpG hypermutation is associated with *TP53* hotspot mutations.**

(**A**) Observed/expected ratio of somatic mutations in trinucleotide contexts of the *TP53* gene. *TP53* mutation data were obtained from the GENIE database. The expected frequency for each mutational context was computed by determining the context for each mutation site and alternate-allele combination registered in the GENIE database, the sum of somatic mutations within each context was then expressed as a proportion of the total number of distinct TP53 mutations in the GENIE database ($n = 1467$). The observed frequency was computed as per the expected, however, each mutational site and the alternate-allele combination was counted once for each sample in which the mutation was observed in the GENIE database. The result was expressed as a proportion of the total number of *TP53* mutations observed in the GENIE database ($n = 39{,}925$). (**B**) Pie-chart of synonymous versus non-synonymous/deleterious SNVs in tumours with the CpG hypermutation phenotype and other somatic hypermutation phenotypes. Colours denote C > T mutations at CpG sites (orange), non-C > T mutations at CpG sites (purple) and all other mutations at non-CpG sites (grey).

© The Author(s)

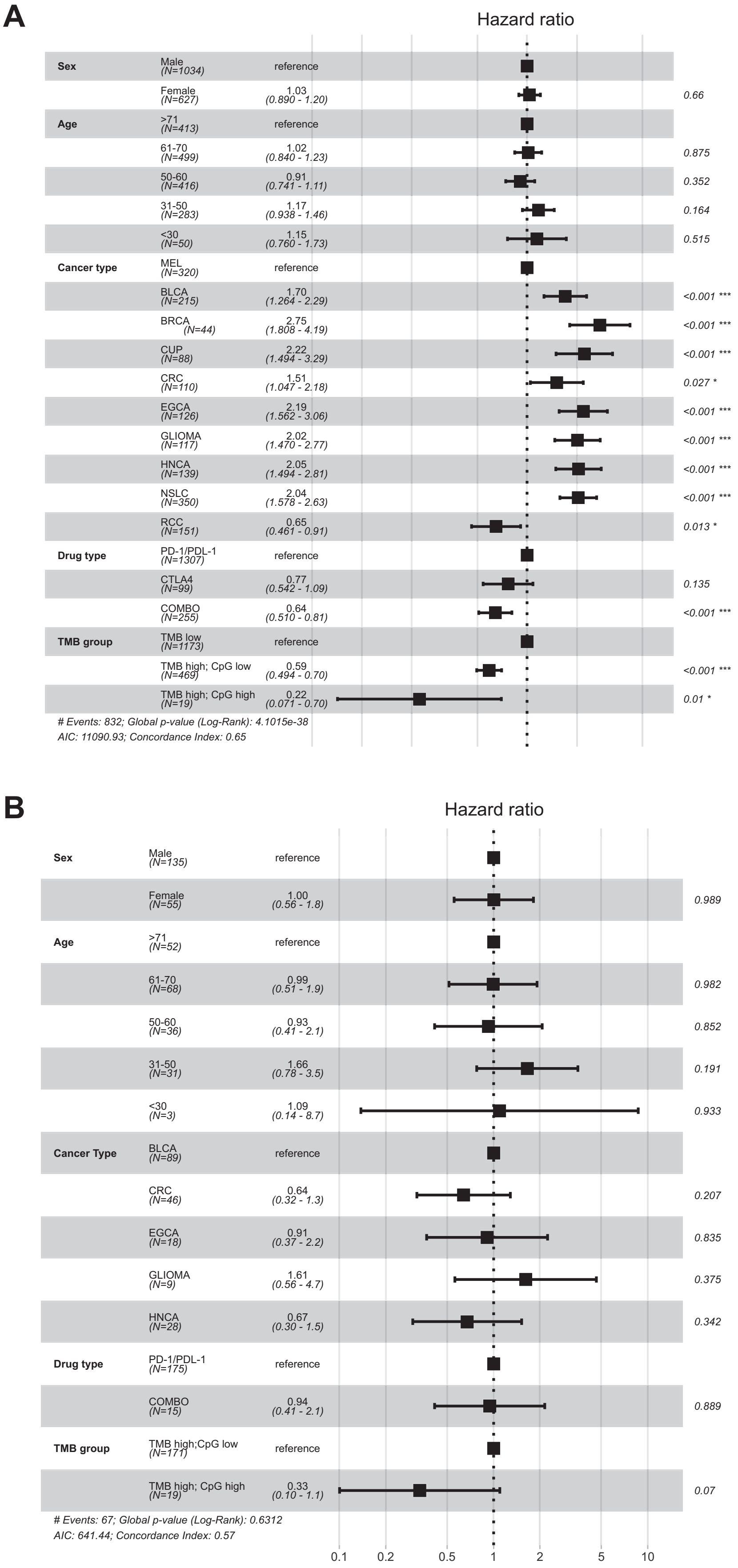
A
Hazard ratio
Sex
Male (N=1034) reference
Female (N=627) 1.03 (0.890 - 1.20) 0.66
Age
>71 (N=413) reference
61-70 (N=499) 1.02 (0.840 - 1.23) 0.875
50-60 (N=416) 0.91 (0.741 - 1.11) 0.352
31-50 (N=283) 1.17 (0.938 - 1.46) 0.164
<30 (N=50) 1.15 (0.760 - 1.73) 0.515
Cancer type
MEL (N=320) reference
BLCA (N=215) 1.70 (1.264 - 2.29) <0.001 ***
BRCA (N=44) 2.75 (1.808 - 4.19) <0.001 ***
CUP (N=88) 2.22 (1.494 - 3.29) <0.001 ***
CRC (N=110) 1.51 (1.047 - 2.18) 0.027 *
EGCA (N=126) 2.19 (1.562 - 3.06) <0.001 ***
GLIOMA (N=117) 2.02 (1.470 - 2.77) <0.001 ***
HNCA (N=139) 2.05 (1.494 - 2.81) <0.001 ***
NSLC (N=350) 2.04 (1.578 - 2.63) <0.001 ***
RCC (N=151) 0.65 (0.461 - 0.91) 0.013 *
Drug type
PD-1/PDL-1 (N=1307) reference
CTLA4 (N=99) 0.77 (0.542 - 1.09) 0.135
COMBO (N=255) 0.64 (0.510 - 0.81) <0.001 ***
TMB group
TMB low (N=1173) reference
TMB high; CpG low (N=469) 0.59 (0.494 - 0.70) <0.001 ***
TMB high; CpG high (N=19) 0.22 (0.071 - 0.70) 0.01 *
# Events: 832; Global p-value (Log-Rank): 4.1015e-38
AIC: 11090.93; Concordance Index: 0.65
B
Hazard ratio
Sex
Male (N=135) reference
Female (N=55) 1.00 (0.56 - 1.8) 0.989
Age
>71 (N=52) reference
61-70 (N=68) 0.99 (0.51 - 1.9) 0.982
50-60 (N=36) 0.93 (0.41 - 2.1) 0.852
31-50 (N=31) 1.66 (0.78 - 3.5) 0.191
<30 (N=3) 1.09 (0.14 - 8.7) 0.933
Cancer Type
BLCA (N=89) reference
CRC (N=46) 0.64 (0.32 - 1.3) 0.207
EGCA (N=18) 0.91 (0.37 - 2.2) 0.835
GLIOMA (N=9) 1.61 (0.56 - 4.7) 0.375
HNCA (N=28) 0.67 (0.30 - 1.5) 0.342
Drug type
PD-1/PDL-1 (N=175) reference
COMBO (N=15) 0.94 (0.41 - 2.1) 0.889
TMB group
TMB high;CpG low (N=171) reference
TMB high; CpG high (N=19) 0.33 (0.10 - 1.1) 0.07
# Events: 67; Global p-value (Log-Rank): 0.6312
AIC: 641.44; Concordance Index: 0.57
0.1
0.2
0.5
1
2
5
10

 © The Author(s)

**Figure EV5. Forest plot for Cox proportional hazards model of overall survival in ICI-treated cancer patients.**

(**A**) Multivariate Cox proportional hazards model for overall survival for all patients. BLCA: $P = 4.5e{-}4$; BRCA: $P = 2.4e{-}6$; CUP: $P = 7.7e{-}5$; EGCA: $P = 5.3e{-}6$; GLIOMA: $P = 1.4e{-}5$; HNCA: $P = 8.6e{-}6$; NSLC: $P = 4.8e{-}8$; COMBO: $P = 1.7e{-}4$; TMB-high;CpG-low: $P = 2.7e{-}9$. Error bars, 95% confidence interval. (**B**) Multivariate Cox proportional hazards model for overall survival for TMB-high patients and cancer types with somatic CpG hypermutators. MEL melanoma, BLCA bladder cancer, BRCA breast cancer, CUP cancer of unknown primary, CRC colorectal cancer, EGCA esophagogastric cancer, GLIOMA glioma, HNCA head and neck cancer, NSCLC non-small cell lung cancer, RCC renal cell carcinoma, TMB tumour mutation burden. Error bars, 95% confidence interval.

© The Author(s)
